# Further thoughts on *P*

**DOI:** 10.3325/cmj.2019.60.570

**Published:** 2019-12

**Authors:** Vladimir Trkulja, Pero Hrabač

**Affiliations:** 1 Department of Pharmacology, Zagreb University School of Medicine, Zagreb, Croatia *vtrkulja@mef.hr*; 2 Department of Medical Statistics, Epidemiology, and Medical Informatics, “Andrija Štampar” School of Public Health, University of Zagreb School of Medicine, Zagreb, Croatia

In the previous issue (1), we addressed the concept of the *P*-value, specifically in the context of the statement by the American Statistical Association (ASA) published in 2016 (2) and the 2019 thematic issue of the American Statistician (3) (by ASA) devoted specifically to this topic. Here, we would like to add a few further comments on the issue, hoping they would be of help to readers who, being statistically lay-persons (like us), might not have time for the in-depth consideration of current developments. For example, a quick overview of the titles of papers accompanying the two ASA documents (eg, *Moving to a World beyond “p<0.05”* and similar) might lead one to assume that the *P*-value concept should be abandoned. The fact is just the opposite – the *P*-value concept has been and remains important for the interpretation of research results (within the frequentist framework). The objective of the entire effort is something else – a deviation that has occurred over the years resulting in a widespread perception of the *P*-value as an ultimate tool for dichotomization of the results as “significant” (typically interpreted as evidence of an effect or, commonly also, as evidence of a “practically relevant effect”) or “not significant” (typically (mis)interpreted as evidence of no effect) based on the magic threshold of 0.05. The entire ASA effort is an attempt to straighten-out this (mal)practice and to endorse (and widely accept) (more) appropriate interpretations. This “call for reason” was also perfectly outlined in a statement published in Nature (4) and signed by more than 800 scientists. In the nine months since it was published, it has been cited 160 times, illustrating the importance that the scientific community attaches to this issue.

The concept of the “critical *P*-value” has been the subject of extensive discussions in the statistical community from its very beginning, some 100 years ago. Already in the 1930s, Pearson had serious reservations about the threshold for statistical significance in the decision process (5). Yet, in the decades to follow, the value of 0.05 as the threshold of “statistical significance” (and, therefore, “evidence”) has become a kind of a dogma in reasoning based on frequentist view on statistics. What is happening at moment is an attempt to demystify it. A critical mass of statistical and other scientists have come together to point out the flaws and poor consequences of many aspects of such practice.

## Where did the 0.05 threshold come from?

In statistics textbooks, lectures, and courses, the value of 0.05 is commonly referred to as the threshold of statistical significance, type I error, or “alpha”. A simplified outline of a typical scenario is that data (numerical values) gathered through an empirical observation (experimental, pseudoexperimental, or any other) are processed by a specific mathematical procedure (statistical test), resulting in a test-statistic and the associated *P*-value. If *P* ≤ 0.05, one concludes that the data are not compatible with the *a priori* hypothesis of “no effect” (the null hypothesis, H_0_), and if *P* > 0.05 one concludes that the data are compatible with H_0_. That is, the *P*-value is considered as a tool for dichotomizing the results of a test. In the former case (*P* ≤ 0.05), one concludes that H_0_ should be rejected; claims the test results to be “statistically significant,” and claims it to be evidence of an effect. In the latter case (*P* > 0.05), one concludes that H_0_ cannot be rejected, considers the test result “not statistically significant,” providing no evidence of an effect. How did this 0.05 threshold come to be in the first place? The common (and basically correct) answer is that this “critical” *P*-value was determined arbitrarily, but with a reason.

The key paper that introduced statistical significance as a concept was that by Edgeworth, published in 1885 on the occasion of the 50th anniversary of the London Royal Statistical Society (6). Edgeworth’s intention was to define a measure that would indicate whether the results of the research deserve further attention or can be ignored. While none of the Fellows of the Society “had Edgeworth’s taste for theory [...] they could see that he was theorising about something worthwhile and that he did it very well” (7). Other authors soon warmed to the concept, concluding that it would be even more useful to have some kind of an indicator that could be used to explicitly decide on the existence or non-existence of statistical significance (8). The term itself was adopted by Fisher (9), who added the concept of null hypothesis. Neyman and E.S. Pearson later extended the concept with type I and type II errors, statistical power, and, critically, with the rejection of the null hypothesis in cases when the *P* value reached the predefined threshold of type I error (10-12).

But, why was the threshold set at 0.05? Karl Pearson (father of the aforementioned E.S. Pearson), in his lectures at University College London, proposed that deviations from the mean that are greater than 3 probable errors were “definitely significant.” (Probable error of the mean (γ) should not be confused with standard deviation (σ); γ = 0.6746 × σ). [A comment: Pearson worked with effects that, as for example difference between two mean values, have a normal sampling distribution. The center, ie, the mean of the normal distribution, is 0 and values that are “3 probable errors” (or more) far from the mean are sufficiently far from it that they could be considered extreme values. Hence, if an observed effect is “3 probable errors” (or more) far from 0, it clearly is not compatible with 0, it is an “extreme” or, as quoted from Pearson, “definitely significant”]. This was embraced by Gosset (better known as “Student”, working in the famous Guinness Brewery and eventually rising to the status of a master brewer) (13). Later, in his seminal 1925 work, Fisher defined more precisely that the total area under the curve of the standard normal distribution that is below and above the mean ±3γ was 4.56%, which he rounded to 5% (8). And thus 0.05. The final step, however, was not made by Fisher, but by Neyman and E.S. Pearson. They introduced the requirement of *a priori* defining this threshold and subsequently basing the decision process (“decision theoretic framework”) on whether the resulting *P* was above or below it. Although the feuds between Neyman and Pearson on the one hand and Fisher on the other were fierce and public, the impression among statisticians was that they agreed about the need to (pre)specify alpha. This fact further cemented the importance of the concept of “statistical significance” among statisticians of the time. The rest, one could say, is history.

## Example (another one...)

The *P*-value has thus become a universal parameter separating statistically significant from not significant, important from irrelevant (to the extremes that went beyond reason and beyond what was the original intention). As Goodman (14), largely ironically, said (on the reasons why it is difficult to “get rid” of this kind of thinking): “When everyone believes in something's value, we can use it for real things; money for food, and *P* values for knowledge claims, publication, funding, and promotion.”

There are numerous examples that illustrate the inappropriateness of decision making solely based on a dichotomized *P*-value. A trivial situation – what if *P* is 0.049, or if *P* is 0.051? Common sense tells us that these values are basically identical. However, the “dichotomization dogma” pushes the conclusion toward “no effect” if *P* = 0.051 and toward “effect” if *P* = 0.049. Furthermore, if this threshold was set arbitrarily at 0.05, why should it not be (also arbitrarily) set at 0.10, 0.15, or 0.02? In other words, what is the value of *P* at which one should “reject the null,” if such a value at all existed? In a meta-analysis of the effects of aerobic exercise on blood pressure (15), authors describe the results of 53 studies, noting that the “effect of exercise” was statistically significant only in a minority of 20 studies. In other words, based on an arbitrary set threshold of statistical significance, 20 studies point to the conclusion that there is a significant blood pressure reduction in those who exercise (an effect of exercise), while the remaining 33 studies find no such reduction (no effect of exercise). However, just an insight into the mean values from two of the embraced studies with such seemingly conflicting results illustrates the pitfalls of “threshold thinking.” In one study, the authors concluded that the change in systolic blood pressure in the exercise group (from 128.6 to 125.3 mm Hg, ie, a reduction of 3.3 mm Hg) was statistically significantly different from the change in control pressure (from 127.6 to 129.9 mm Hg, ie, an increase by 2.3 mm Hg, for an overall difference in mean change of 5.6 mm Hg) (16). In another study, the authors did not find statistically significant differences between the exercise group (change in SBP from 136.6 to 130.1 mm Hg, ie, a 5.5 mm Hg reduction) and the control group (change from 134.9 to 135.8 mm Hg, ie, a 0.9 mm Hg increase, for an overall mean difference of 6.4 mm Hg) (17). Observing only the blood pressure values, ie, the effect size, it is clear that both studies point to exactly the same conclusion. To imply based on the results of the latter study that there is no (beneficial) effect of physical activity on blood pressure is completely meaningless. This is an error commonly seen in various publications, arising from ignoring the effect size, results of other similar studies, and treating *P*-value as an uttermost indicator of truthfulness of the underlying concept. *P*-value is actually a measure of the compatibility of the observed effect with the *a priori* null hypothesis, which communicates uncertainty about the effect: low *P*-values signal poor compatibility with the null and increase our certainty about the existence of an effect, while high *P*-values signal better compatibility with the null and increase our uncertainty about the existence of an effect.

## So, what to do? Abandon *P*, change threshold?

Should the *P*-value then be ignored? Definitely not. However, its interpretation should be more flexible, as opposed to the “all or nothing” threshold-based reasoning. In the above examples, in the first study *P* was <0.001, while in the second study it was 0.11. Deciding (just) based on *P* would have led us in the wrong direction, but looking at the effect size and placing the results in the context of other studies points to a correct conclusion. So, the correct question is not what metric to use instead of *P*, but how to interpret *P* and how to best supplement it with other indicators.

Some hints regarding this approach could be summarized as follows:

• When postulating a scientific question or hypothesis, multiple studies should be used to answer one question (the concept of “one phenomenon, many studies”) rather than trying to answer many questions in a single study (the concept of “many phenomena, one study”) (18).

• The data gathering process should always be evaluated first, eg, to try to assess to what level it has been protected from various types of bias and confounding – ie, to ascertain that the observed effect indeed could be ascribed to the intervention (treatment, risk factor, diagnostic test, etc) studied.

• The mathematical method used to “process the data” (ie, the method of effect calculation) should be appropriate and reported in detail. In other words – potential bias arising from data gathering process and data processing should be minimized.

• The *P*-value should be interpreted in the context of the sample size and the observed effect size and should be always reported with the estimated effect and its confidence intervals (19).

• The results should never be described solely as statistically significant or not significant based on some arbitrary threshold. *P* should be treated as a “continuum of compatibility with the null hypothesis,” reported accurately to three decimal places. It has been suggested that the wording “statistically significant” should be used only in conjunction with very low *P* values (<0.005) (20), ie, that the threshold should be reduced – but this is a matter of debate. For example, in pivotal regulatory trials of therapeutics, which are all stringently conducted experiments, *P* < 0.05 has been continuously considered as a sufficient level of certainty about the existence of an effect – and the concept seems to function well.

• The fact is that all empirical observations are done in samples from the population – and the only constant in life sciences and medicine is a great variability of the studied entities. Therefore, it is highly unlikely that all studies of a particular phenomenon will produce the same results. Results will vary, and so will the values of obtained parameters, statistical and otherwise. Only a shift from statistical inference to (much more demanding) statistical thinking allows us to draw the right conclusions based on a large amount of information.
